# The predictors of depression and burnout among surgical residents: A cross-sectional study from Kuwait

**DOI:** 10.1016/j.amsu.2021.102337

**Published:** 2021-04-21

**Authors:** Waleed Burhamah, Abdulaziz AlKhayyat, Melinda Oroszlányová, Hana Jafar, Ali AlKhayat, Jasim Alabbad

**Affiliations:** a Department of General Surgery, Mubarak AlKabeer Hospital, Kuwait; b Ministry of Health, Kuwait; c College of Engineering and Technology, American University of the Middle East, Kuwait

**Keywords:** Surgery, Surgical residency, Burnout, Depression, Surgeon wellbeing, Mental health

## Abstract

**Background:**

Surgical residency often poses a challenge to residents, with long working hours and a stressful work environment. Surgical residents are at an increased risk of burnout and depression. Such mental health burdens could go so far as to affect treatment outcomes.

**Aim:**

To assess the prevalence and risk factors for depression and burnout among residents across surgical specialties in Kuwait.

**Materials and methods:**

An online questionnaire was sent to the residents enrolled to the surgical residency programs in Kuwait, from the period of January 2020–February 2020. Variables collected included; age, gender, marital status, smoking history, exercise, specialty, year of training, on-call frequency, assessment of burnout (using the abbreviated Maslach Burnout Inventory (aMBI)) and assessment of depressive symptoms (using the Patient Health Questionnaire-9 (PHQ-9) score).

**Results:**

A total of 85 surgical residents between the age of 20 and 40 years responded. Most (64.7%) were male and 35.3% female. More than half were married (51.8%) and 41.2% were single. The majority of the residents were in general surgery (43.5%), with the least being in otolaryngology (7.1%) and neurosurgery (5.9%). The prevalence of depressive symptoms was 55.3%, and 51.8% had a high overall burnout score.

**Conclusion:**

Addressing burnout at all stages during residency training is paramount in improving standard of care as well as increasing the wellness of residents.

## Highlights

• Residents are a population at high risk for burnout and depression.
• Such mental burdens could go so far as to affect treatment outcomes.
• The prevalence for depression and burnout among residents across surgical specialties in Kuwait is high.
• Many risk factors have been identified and are comparable to the existing literature.
• Addressing burnout at all stages during residency programs is paramount in improving standard.

## 1 Introduction

Surgical residency often poses a challenge to residents; with long, unpredictable working hours, a stressful work environment and high-stake surgeries on an almost daily basis. Recent data suggest that such mental burdens could go so far as to affect treatment outcomes [[1], [2], [3]].

Burnout is defined as a state of emotional, physical and mental exhaustion caused by excessive and prolonged stress. Risk factors include long working hours, high stress levels and sleep deprivation [7]. It has been linked to an increased rate of substance abuse, poor interpersonal and vocational functioning, as well as low job satisfaction [4]. Unfortunately burnout is prevalent among surgical residents, with some studies showing rates up to 76% of the participants [5,6]. Burnout has recently grown to become a public health concern, as it appears to increase the risk of depression [8,9], anxiety [9] and suicidality [8,9].

Various measures have been implemented to help shed light on the issue of burnout, such as lectures teaching residents skills to help combat burnout and prioritize mental well-being [11,12]. Additionally, the American Council for Graduate Medical Education (ACGME) implemented new rules regarding working hours; residents are not to exceed a maximum of 24 h of continuous duty. Yet it is unclear if such interventions have been successful in decreasing burnout among surgical residents [13,14].

Aim: To assess the prevalence and identify the predictors of depression and burnout among residents across all surgical specialties in Kuwait.

## 2 Methods

### 2.1 Participants and survey design

This is an online questionnaire based cross-sectional study conducted in Kuwait from January 2020–February 2020. An online questionnaire was sent to residents enrolled in the surgical residency programs in Kuwait. Residency training in Kuwait provides programs in both medical and surgical specialties. Specialties included in our cohort were: general surgery, orthopaedic surgery, urology, neurosurgery, obstetrics and gynaecology and otolaryngology. An email was sent to our participants, it included an invitation letter detailing the reason behind the questionnaire and that it is confidential and anonymous. Consent was obtained from participants. Variables collected included; age, gender, marital status, smoking history, exercise, specialty, year of training, on call frequency, assessment of burnout and assessment of depressive symptoms. Ethical approval was obtained from the ethical committee in the Ministry of Health, Kuwait. The study is registered on ClinicalTrials.gov under the unique identity number NCT04808635 [50], and is reported in line with the STROCSS criteria [49].

### 2.2 Assessment of depression

Symptoms of depression were assessed using the 9-item Patient Health Questionnaire (PHQ-9) score, a self-report questionnaire that is commonly used as a screening tool for depression [15]. It is composed of 9 questions assessing the frequency of depressive symptoms on a 4-point liker-scale ranging from 0 (never) to 3 (nearly every day). The total score was calculated for each participant and was interpreted as follows: minimal (1–4), mild (5–9), moderate (10–14), moderately severe (15–21), severe (20–27). A standard cut-off score of 10 was used, indicating a diagnosis of depression [16,17].

### 2.3 Assessment of burnout

To measure burnout we used the abbreviated 9-item form of the Maslach Burnout Inventory - Human Services Survey (aMBI-HSS) [18]. The aMBI is a 9-item questionnaire, where each question is rated on a 7-point liker-scale ranging from 0 to 6, which indicates the frequency of symptoms in question. Response options include; ‘Every day’, ‘A few times a week’, ‘Once a week’, ‘A few times a month’, ‘Once a month’, ‘A few times a year’, or ‘Never’. The questions are grouped into 3 subscales (emotional exhaustion (EE), depersonalization (DP) and personal accomplishment (PA)) and are evaluated and scored separately with each score ranging from 0 to 18. We used the cut-off points set by Lebares et al. for EE and D. The cut-off points for high burnout among U.S. surgery residents were: EE score ≥9 and DP score ≥6. A PA score ≤12 was chosen as a cut-off for high burnout.

### 2.4 Statistical analysis

The data analysis was performed on 85 responders using R software, version 3.6.3. The categorical variables were expressed as frequencies and percentages, and the continuous variables were expressed as mean and standard deviation (SD). The categorical variables were tested by using the Chi-squared test (at α = 0.05), and the standardized residuals (SR) were calculated. The continuous variables were tested by the *t*-test, Mann-Whitney test and Kruskal-Wallis test (at α = 0.05), and the point-biserial correlation coefficients ($r_{pb}$) were calculated. Multivariable logistic regression was performed to identify the potential risk factors for depression and burnout in surgical residents, using the statistically significant variables (|SR| > 2, $r_{pb}>0.5$). We adjusted for confounders and calculated the odds ratios (OR), the 95% confidence intervals (CI) and the corresponding p-values.

## 3 Results

The demographic characteristics of the responding surgical residents are summarized in Table 1. Out of the total 85 responders between the age of 20 and 40 years, 55 (64.7%) were male and 46 (35.3%) female, more than half being married (51.8%) and 41.2% single. Most of the residents were between 26–30 years old (61.2%), and 34.1% were in the age category of 31–35 years old. Majority of the surgical residents were smokers (68.2%), exercise regularly (61.2%) and have no medical conditions (76.5%). Most of the residents were in general surgery (43.5%), with the least being in urology (10.6%), otolaryngology (7.1%) and neurosurgery (5.9%). First-year residents (R1) made up the largest proportion of the responders (44.7%), 18.8% were in their third year (R3), 12.9% were in their second year (R2), 12.9% were fifth-year residents (R5) and 10.6% were fourth-year residents (R4). The majority of the surgical residents were on call every 4th day (68.2%), 27.1% were on call only every 5th day, and only 4.7% were on call every 3rd day.

**Table 1:** Demographic characteristics of responding surgical residents.

Variable	All cases (n = 85 (%))
Gender	
Female	30 (35.3)
Male	55 (64.7)
Marital status	
In a relationship	5 (5.9)
Married	44 (51.8)
Separated/divorced	1 (1.2)
Single	35 (41.2)
Age	
20-25	2 (2.4)
26-30	52 (61.2)
31-35	29 (34.1)
36-40	2 (2.4)
Smoker	
No	58 (68.2)
Yes	27 (31.8)
Exercising regularly	
No	52 (61.2)
Yes	33 (38.8)
Medical condition	
No	65 (76.5)
Yes	20 (23.5)
Specialty	
Otolaryngology	6 (7.1)
General surgery	37 (43.5)
Neurosurgery	5 (5.9)
Obstetrics and gynaecology	14 (16.5)
Orthopaedic surgery	14 (16.5)
Urology	9 (10.6)
Year	
R1	38 (44.7)
R2	11 (12.9)
R3	16 (18.8)
R4	9 (10.6)
R5	11 (12.9)
On call frequency	
Every 3rd day	4 (4.7)
Every 4th day	58 (68.2)
Every 5th day	23 (27.1)
Depression	
Mild	31 (36.5)
Moderate	31 (36.5)
Moderately severe	13 (15.3)
None	7 (8.2)
Severe	3 (3.5)
Depression 10 or more	
No	38 (44.7)
Yes	47 (55.3)
Total PHQ9 score*	10.2 (4.7)
Total PA*	13.9 (3.6)
Total EE*	10.6 (4.1)
Total DP*	4.4 (4.1)
Total burnout*	15 (6.9)

*Mean (SD).

The prevalence of depressive symptoms (i.e. having a PHQ-9 score of 10 or more) was 55.3%, more than half of our residents. Mild depression was observed in 36.5% and moderate depression in 36.5% of the residents. Another 15.3% had moderately severe depression, and 3.5% had severe depression. The remaining 8.2% of the residents had no symptoms of depression. The average PHQ9 score was 10.2, and the average personal accomplishment (PA) score was 13.9. For emotional exhaustion (EE), the average score was 10.6, and for depersonalization (DP) 4.1. The average score for burnout was 15.

The average personal achievement score (PA) was less for depressed residents (13.6) than for residents without depression (14.2). The average scores for emotional exhaustion (12.2), depersonalization (5.0) and burnout (17.2) were higher for residents with depression than for the residents who were not depressed (8.6, 3.6 and 12.2, respectively).

The data shown in Table 2 reveal that 55.3% of surgical residents are depressed. Exactly half of the female residents (50.0%) and more than half of the male residents (58.2%) had depression. Smokers tend to be more depressed (59.3%) than non-smokers (53.4%). Residents exercising on a regular basis are much less depressed (33.3%) than residents who do not exercise regularly (69.2%). Residents with a medical condition have a higher tendency for depression (65.0%) than residents without a medical condition (52.3%). Residents in obstetrics and gynaecology (64.3%), general surgery (62.2%), neurosurgery (60.0%) and urology (55.6%) tend to be more depressed than residents in orthopaedic surgery (42.9%) and otolaryngology (16.7%). The first-year residents tend to be less depressed (47.4%) than the second to fifth-year residents (50.0%–81.8%).

**Table 2:** Factors associated with depression amongst responding surgical residents.

Variable	n (%)	No depression	Depression	P-value
Total	85 (100)	38 (44.7)	47 (55.3)	
Gender				
Female	30 (35.3)	15 (50.0)	15 (50.0)	0.619
Male	55 (64.7)	23 (41.8)	32 (58.2)	
Marital status				
In a relationship	5 (5.9)	2 (40.0)	3 (60.0)	0.786
Married		44 (51.8)	21 (47.7)	23 (52.3)
Separated/divorced	1 (1.2)	0 (0)	1 (100.0)	
Single	35 (41.2)	15 (42.9)	20 (57.1)	
Age				
20-25	2 (2.4)	1 (50.0)	1 (50.0)	0.429
26-30	52 (61.2)	26 (50.0)	26 (50.0)	
31-35	29 (34.1)	11 (28.9)	18 (38.3)	
36-40	2 (2.4)	0 (0)	2 (100.0)	
Smoker				
No	58 (68.2)	27 (46.6)	31 (53.4)	0.789
Yes	27 (31.8)	11 (40.7)	16 (59.3)	
Exercising regularly				
No	52 (61.2)	16 (30.8)	36 (69.2)	0.003
Yes	33 (38.8)	22 (66.7)	11 (33.3)	
Medical condition				
No	65 (76.5)	31 (47.7)	34 (52.3)	0.459
Yes	20 (23.5)	7 (35.0)	13 (65.0)	
Specialty				
Otolaryngology	6 (7.1)	5 (83.3)	1 (16.7)	0.336
General surgery	37 (43.5)	14 (37.8)	23 (62.2)	
Neurosurgery	5 (5.9)	2 (40.0)	3 (60.0)	
Obstetrics and gynaecology	14 (16.5)	5 (35.7)	9 (64.3)	
Orthopaedic surgery	14 (16.5)	8 (57.1)	6 (42.9)	
Urology	9 (10.6)	4 (44.4)	5 (55.6)	
Year				
R1	38 (44.7)	20 (52.6)	18 (47.4)	0.314
R2	11 (12.9)	5 (45.5)	6 (54.5)	
R3	16 (18.8)	8 (50.0)	8 (50.0)	
R4	9 (10.6)	3 (33.3)	6 (66.7)	
R5	11 (12.9)	2 (18.2)	9 (81.8)	
On call frequency				
Every 3rd day	4 (4.7)	0 (0)	4 (100.0)	0.170
Every 4th day	58 (68.2)	28 (48.3)	30 (51.7)	
Every 5th day	23 (27.1)	10 (43.5)	13 (56.5)	
Total PHQ9 score*	10.2 (4.7)	6.2 (2.1)	13.4 (3.7)	<0.001
Total PA*	13.9 (3.6)	14.2 (3.7)	13.6 (3.5)	0.283
Total EE*	10.6 (4.1)	8.6 (4.4)	12.2 (3.1)	<0.001
Total DP*	4.4 (4.1)	3.6 (3.9)	5.0 (4.3)	0.105
Total burnout*	15 (6.9)	12.2 (6.8)	17.2 (6.2)	0.001

*Mean (SD).

The variables listen in Table 2 were tested individually in relation to depression. The tests showed statistically significant negative association between depression and regular exercising (SR = −1.7, p-value = 0.003). The results also showed a statistically significant difference between the average PHQ9 score, emotional exhaustion, and burnout for depressed and non-depressed residents (p-value < 0.01), and statistically significant associations ($r_{pb}$ = 0.8, $r_{pb}$ = 0.4, $r_{pb}$ = 0.4, respectively). The multivariable logistic regression analysis, including the variables that showed statistically significant association with depression, emphasizes the influence of regular exercising and emotional exhaustion on depression (p-value < 0.05). The odds ratios and confidence intervals are presented in Table 3.

**Table 3:** Multivariable analyses of factors influencing depression amongst responding surgical residents.

Variable	P-value	OR	C.I. (95%)
Exercising regularly	0.0005	0.12	0.03–0.37
Total emotional exhaustion	0.0314	1.33	1.03–1.76

Table 4 presents the prevalence of burnout characteristics amongst surgical residents. We can observe that 72.9% of the responders scored high in emotional exhaustion, 38.8% scored high in depersonalization, 30.6% scored low in personal achievement and 51.8% scored high in overall burnout. Table 5 compares aMBI constructs with the demographic characteristics of responding surgical residents. We can observe that most of the characteristics have a similar average between their categories by every construct.

**Table 4:** Prevalence of burnout characteristics amongst responding surgical residents.

Characteristic	n (%)
Emotional Exhaustion (EE)	
Low (<9)	23 (27.1)
High (>= 9)	62 (72.9)
Depersonalization (DP)	
Low (<6)	52 (61.2)
High (>= 6)	33 (38.8)
Personal Achievement (PA)	
Low (<= 12)	26 (30.6)
High (>12)	59 (69.4)
Burnout (EE + DP)	
Low (<15)	41 (48.2)
High (≥ 15)	44 (51.8)

**Table 5:** Comparison of aMBI constructs by demographic characteristics of responding surgical residents

Variable			Emotional exhaustion			Depersonalization			Personal accomplishment			Burnout		
		N (%)	Mean	Median	SD	Mean	Median	SD	Mean	Median	SD	Mean	Median	SD
Total		85 (100)	10.6	10.0	4.1	4.4	3.0	4.1	13.9	15.0	3.6	15.0	15.0	6.9
Gender														
Female		30 (35.3)	10.3	10.0	3.9	3.7	3.5	3.0	14.3	15.0	2.9	14.0	14.5	6.3
Male		55 (64.7)	10.8	11.0	4.3	4.7	3.0	4.6	13.6	15.0	4.0	15.5	15.0	7.2
	**p-value**		**0.344**			**0.742**			**0.795**			**0.350**		
Marital status														
In a relationship		5 (5.9)	10.4	12.0	5.2	4.6	3.0	0.6	15.4	12.0	3.1	15.0	15.0	2.5
Married		44 (51.8)	10.2	10.0	4.5	3.8	3.0	3.8	14.0	12.8	3.6	14.0	13.0	7.4
Separated/divorced		1 (1.2)	12.0	12.0	NA	3.0	3.0	NA	11.0	11.0	NA	15.0	15.0	NA
Single		35 (41.2)	11.1	10.0	3.7	5.1	6.0	4.6	13.5	10.5	3.8	16.2	16.0	6.7
	**p-value**		**0.899**			**0.738**			**0.447**			**0.623**		
Age														
20-25		2 (2.4)	9.0	9.0	0.0	7.0	7.0	2.8	12.5	12.5	4.9	16.0	16.0	2.8
26-30		52 (61.2)	10.3	10.0	4.3	4.1	3.0	4.2	13.8	15.0	3.7	14.4	14.0	7.0
31-35		29 (34.1)	11.2	11.0	4.1	4.7	5.0	4.2	13.9	15.0	3.6	16.0	17.0	7.3
36-40		2 (2.4)	12.0	12.0	4.2	3.0	3.0	4.2	16.0	16.0	1.4	15.0	15.0	0.0
	**p-value**		**0.567**			**0.522**			**0.818**			**0.567**		
Smoker														
No		58 (68.2)	10.5	10.0	4.2	4.4	3.0	4.3	14.1	15.0	3.8	14.9	15.0	7.4
Yes		27 (31.8)	10.9	11.0	4.0	4.3	5.0	3.9	13.5	14.0	3.3	15.2	15.0	5.8
	**p-value**		**0.680**			**0.977**			**0.296**			**0.762**		
Exercising regularly														
No		52 (61.2)	10.5	10.0	3.9	4.0	3.0	4.0	14.1	15.0	3.3	14.6	15.0	6.8
Yes		33 (38.8)	10.7	11.0	4.6	4.9	4.0	4.4	13.5	15.0	4.1	15.6	15.0	7.2
	**p-value**		**0.807**			**0.469**			**0.696**			**0.570**		
Medical condition														
No		65 (76.5)	10.8	11.0	4.0	4.5	3.0	4.0	13.8	15.0	3.8	15.3	15.0	7.4
Yes		20 (23.5)	10.1	10.0	3.6	3.9	3.5	3.2	14.1	15.0	3.2	13.9	13.0	5.0
	**p-value**		**0.336**			**0.742**			**0.766**			**0.458**		
Specialty														
Otolaryngology		6 (7.1)	6.7	8.0	3.4	2.0	0.5	3.2	14.0	16.5	5.7	8.7	8.5	5.9
General surgery		37 (43.5)	11.4	11.0	4.0	5.0	5.0	4.3	13.6	15.0	3.9	16.4	15.0	7.3
Neurosurgery		5 (5.9)	14.0	15.0	4.2	6.4	6.0	4.6	15.6	16.0	1.7	20.4	21.0	5.9
Obstetrics and gynaecology		14 (16.5)	8.9	9.5	3.1	2.7	2.0	2.7	15.6	15.5	1.7	11.6	11.5	5.3
Orthopaedic surgery		14 (16.5)	9.6	11.0	4.9	3.5	1.0	4.5	12.8	1.5	3.1	13.1	12.5	6.0
Urology		9 (10.6)	12.6	12.0	2.5	5.9	5.0	4.0	12.8	15.0	4.1	18.4	19.0	4.2
	**p-value**		**0.009**			**0.115**			**0.232**			**0.004**		
Year														
R1		38 (44.7)	9.4	9.0	3.8	3.6	3.0	3.7	14.6	16.0	3.2	13.0	13.5	6.3
R2		11 (12.9)	12.5	12.0	3.6	5.5	3.0	5.6	12.7	15.0	5.2	18.0	16.0	8.0
R3		16 (18.8)	11.2	12.0	4.4	5.1	5.5	3.5	12.9	14.0	3.2	16.3	17.5	5.7
R4		9 (10.6)	11.6	11.0	4.5	4.2	6.0	3.7	14.1	15.0	3.7	15.8	15.0	6.7
R5		11 (12.9)	11.2	12.0	4.7	4.8	3.0	5.2	13.7	15.0	3.8	16.0	15.0	8.7
	**p-value**		**0.130**			**0.601**			**0.430**			**0.255**		
On call frequency														
Every 3rd day		4 (4.7)	14.5	15.0	1.7	6.0	4.5	6.5	14.5	15.5	2.4	20.5	18.5	6.9
Every 4th day		58 (68.2)	10.8	11.0	4.3	4.3	3.5	3.9	13.4	15.0	3.9	15.1	15.0	6.7
Every 5th day		23 (27.1)	9.6	10.0	3.6	4.2	3.0	4.4	15.0	16.0	2.8	13.8	14.0	7.1
	**p-value**		**0.039**			**0.888**			**0.223**			**0.225**		
Depression														
Mild		31 (36.5)	9.3	9.0	4.1	3.5	3.0	3.8	14.0	15.0	4.0	12.8	12.0	6.9
Moderate		31 (36.5)	11.7	12.0	2.8	3.8	4.0	3.6	14.0	15.0	3.1	15.5	16.0	5.0
Moderately severe		13 (15.3)	12.5	13.0	3.5	5.9	6.0	3.8	12.8	13.0	4.0	18.5	18.0	5.8
None		7 (8.2)	5.6	4.0	4.8	3.6	1.0	4.4	14.9	16.0	2.5	9.1	9.0	5.8
Severe		3 (3.5)	16.3	16.0	1.5	13.3	14.0	2.1	12.7	16.0	6.7	29.7	30.0	2.5
	**p-value**	** **	**0.0003**	** **	** **	**0.020**	** **	** **	**0.784**	** **	** **	**0.0005**		

The surgical specialty showed significant difference in emotional exhaustion (p-value = 0.009) and overall burnout (p-value = 0.004). On call frequency correlated significantly with emotional exhaustion (p-value = 0.039). While depression showed a significant difference in emotional exhaustion (p-value = 0.0003), depersonalization (p-value = 0.020) and overall burnout (p-value = 0.0005).

Applying the thresholds for the aMBI constructs, we can see in Table 6 that all responding surgical residents showed high level of emotional exhaustion (>50%), regardless of their gender, marital status, age, medical history, smoking and exercising habits, specialty, resident-years and on call frequency, except residents with no depression (29%).

**Table 6:** Factors associated with burnout amongst responding surgical residents.

Variable	n (%)	EE≥9	DP≥6	PA≤12	EE + DP≥15
Total	85 (100)	62 (72.9)	33 (38.8)	26 (30.6)	44 (51.8)
Gender					
Female	30 (35.3)	18 (60)	10 (33)	7 (23)	15 (50)
Male	55 (64.7)	44 (80)	23 (42)	19 (35)	29 (53)
Marital status					
In a relationship	5 (5.9)	4 (80)	1 (20)	2 (40)	3 (60)
Married	44 (51.8)	30 (68)	14 (32)	11 (25)	19 (43)
Separated/divorced	1 (1.2)	1 (100)	0 (0)	1 (100)	1 (100)
Single	35 (41.2)	27 (77)	18 (51)	12 (34)	21 (60)
Age					
20-25	2 (2.4)	2 (100)	1 (50)	1 (50)	1 (50)
26-30	52 (61.2)	35 (67)	18 (35)	16 (31)	25 (48)
31-35	29 (34.1)	23 (79)	13 (45)	9 (31)	16 (55)
36-40	2 (2.4)	2 (100)	1 (50)	0 (0)	2 (100)
Smoker					
No	58 (68.2)	39 (67)	21 (36)	17 (29)	30 (52)
Yes	27 (31.8)	23 (85)	12 (44)	9 (33)	14 (52)
Exercising regularly					
No	52 (61.2)	38 (73)	19 (37)	15 (29)	27 (52)
Yes	33 (38.8)	24 (73)	14 (42)	11 (33)	17 (52)
Medical condition					
No	65 (76.5)	49 (75)	25 (38)	18 (28)	36 (55)
Yes	20 (23.5)	13 (65)	8 (40)	8 (40)	8 (40)
Specialty					
Otolaryngology	6 (7.1)	3 (50)	1 (17)	1 (17)	1 (17)
General surgery	37 (43.5)	30 (81)	17 (46)	14 (38)	21 (57)
Neurosurgery	5 (5.9)	4 (80)	4 (80)	0 (0)	4 (80)
Obstetrics and gynaecology	14 (16.5)	8 (57)	3 (21)	1 (7)	5 (36)
Orthopaedic surgery	14 (16.5)	8 (57)	4 (29)	7 (50)	5 (36)
Urology	9 (10.6)	9 (100)	4 (44)	3 (33)	8 (89)
Year					
R1	38 (44.7)	24 (63)	12 (32)	9 (24)	16 (42)
R2	11 (12.9)	10 (91)	4 (36)	4 (36)	7 (64)
R3	16 (18.8)	12 (75)	8 (50)	7 (44)	10 (63)
R4	9 (10.6)	7 (78)	5 (56)	1 (11)	5 (56)
R5	11 (12.9)	9 (82)	4 (36)	5 (45)	6 (55)
On call frequency					
Every 3rd day	4 (4.7)	4 (100)	2 (50)	1 (25)	4 (100)
Every 4th day	58 (68.2)	43 (74)	23 (40)	20 (34)	30 (52)
Every 5th day	23 (27.1)	15 (65)	8 (35)	5 (22)	10 (43)
Depression					
Mild	31 (36.5)	19 (61)	9 (29)	8 (26)	11 (35)
Moderate	31 (36.5)	27 (87)	11 (35)	9 (29)	20 (65)
Moderately severe	13 (15.3)	11 (85)	8 (62)	6 (46)	9 (69)
None	7 (8.2)	2 (29)	2 (29)	2 (29)	1 (14)
Severe	3 (3.5)	3 (100)	3 (100)	1 (33)	3 (100)

We can also observe in Table 6 that male residents were emotionally more exhausted than female residents, they also scored higher in depersonalization and overall burnout. Smokers were emotionally more exhausted, feeling more depersonalized than non-smoking residents. Residents in urology and neurosurgery were the most emotionally exhausted and feeling the most depersonalized with a higher overall burnout score. Finally the residents with severe or moderately severe depression tend to feel the most emotionally exhausted, depersonalized and burned out.

The variables listen in Table 6 were tested one by one in relation to burnout. There was a statistically significant association between depression (p-value = 0.009) and specialty (p-value = 0.026), and overall burnout, respectively.

## 4 Discussion

Surgical specialties are known to be amongst the most demanding fields in medical practice. In a systemic review by Low et al. [19] the prevalence of burnout among surgical residents was found to be as high as 51%. The depersonalization dimension of burnout has been shown to be associated with lower patient satisfaction and a longer recovery time [20]. Stigma surrounding the stress and burnout associated with surgical specialties has also proven to be a deterrent to medical students in the US; resulting in a decline in applications to surgical residency programs. Primary deterrents have been identified as the lifestyle of a surgeon, stress, inadequate personal and family time and uncertainty regarding future income [26,27]. This provides another reason why more proactive steps should be taken to address burnout. The literature provides strong evidence on several risk factors for higher levels of burnout and psychological distress among surgical residents [[21], [22], [23]]. However interestingly, higher rates of emotional intelligence were associated with lower burnout rates [24,25].

In our study we quantify the prevalence of burnout and depression among residents across all surgical fields in Kuwait. We further identify potential risk factors; this allows us to determine the need for further interventions to address a worldwide issue facing surgeons in training. In our experience the prevalence of burnout was found to be 51.8%, similar to what was reported by Low et al. [19]. However, our prevalence was found to be lower than data from neighboring countries [8,[28], [29], [30], [31]]. In several studies, on-call frequency was found to be one of the main risk factors for burnout, and Kuwait's surgical residency programs generally had less on-calls than residencies in the aforementioned studies.

Albeit a small difference, our study showed a higher prevalence of burnout in males (53% Vs 50%). In a systematic review examining the effect of gender and burnout, 6 articles showed that females have higher burnout levels, 1 in favor of males and 9 studies showed no significant different [21]. Galaiya et al. [22] concluded 11 studies showed higher burnout levels in females and 3 studies showed higher levels in males. It seems that in general, most data suggests that females are more prone to burnout during surgical residency [30,32,33]. Factors contributing to a higher prevalence of burnout among female surgical residents when compared to their male counterparts can be due to certain means of mistreatment such as discrimination, abuse and harassment. The rates of mistreatment were found to be higher among female surgical residents, with up to 65.1% reporting gender discrimination and 19.9% reporting sexual harassment. Attending surgeons were found to be the most frequent sources of abuse (51.9%).

Younger residents and those with junior status were more likely to be burnt out. This is supported by several other studies and systematic reviews [22,23,33]. Though not statistically significant, in our experience burnout scores were shown to be higher in the age groups between 20 and 25. Age 20–25 is when most surgical residents in Kuwait are beginning their residency training. Adjusting to the longer hours, increased responsibility and high expectations associated with residency. This can explain this trend. Interestingly, senior residents in their R5 year were shown to have one of the lowest levels of burnout amongst the training years.

Furthermore marriage was associated with less burnout rates and lower overall burnout scores, and being single was associated with higher scores. This can be due to the higher social support provided by a spouse and is supported by a number of studies [22,23,28,[32], [33], [34]]. One study [30] showed no correlation in relationship status with levels of burnout – but found that having a child was associated with a higher personal achievement score.

In our experience exercise seemed to play no role in lowering burnout rates, and paradoxically, though not statistically significant – those who exercised regularly had higher burnout scores than those who did not (15.6 > 14.6). However, the literature showed significantly lower levels of burnout in those who exercised regularly [8,22,35,36]. Hence organizing regular group sports activities, such as marathons and sports tournaments, and promoting a healthier more active lifestyle could be potential interventions programs should implement.

Otolaryngology residents had both the lowest burnout rates and overall burnout scores (17%, 8.7 respectively). A similar finding was observed in multiple previous studies [21,[37], [38], [39], [40], [41], [42], [43], [44]]. Urology residents showed the highest prevalence of burnout (89%), however, the highest mean overall burnout score was that of neurosurgical residents (20.4). Year of training was also a significant factor. Second year of training, commonly referred to as R2, exhibited both highest burnout scores and prevalence (18, 64% respectively). This is supported by multiple studies [22,29,35,45]. R2 is the year in which residents commence taking on a more senior role, having to overlook the newly recruited residents into the training program and ensure work in done at the standard expected. This leads to a higher shift of ergonomic burden and increased expectations by their superiors.

Several interventions can potentially reduce burnout rates. Raising awareness, mindfulness, organizing social events, wellness activities with faculty members were all associated with lower rates of burnout and depression [46].

Along with burnout, depression is another issue facing surgeons in training. In a systematic review by Oskrochi et al. [23], rates of depression among surgical residents was reported to be between 30.8 and 37.5%, lower that what was seen in our study. We had an overall prevalence of 55%. Factors associated with a higher risk of depression were male gender, regular smoking status, lack of exercise, those with medical conditions, being in obstetrics and gynaecology residency, being unmarried, being oncall every 3rd day and approaching end of residency training (year R5).

Exercise is known to be an effective tool in combatting anxiety, depression and other mood disorders [47], so it is of no surprise that less depression was found in those who exercised regularly. A busier oncall schedule limits the resident's time to engage in distressing activities and hobbies. A study undertaken in Oman [48] showed a higher likelihood of depression in females, junior and non-exercising residents. Regularly exercising residents had a 21.8% prevalence of mild to moderate depression, but interestingly – 0% of severe depression. Another study undertaken in Stanford, CA [10] showed higher predispositions of single individuals to depression than those who are married; however no significant difference in postgraduate level.

In our experience there was a significant association between higher levels of burnout in depressed residents. Depressed residents were found to have higher emotional exhaustion and depersonalization than those who were not depressed; while personal achievement scores were also lower in depressed residents. This supports the data available in recent literature, where burnout has been shown to increase the risk of depression [8,9], anxiety [9] and suicidality [8,9]. Reinforcing the need to address the issue of burnout in residency training.

### 4.1 Limitations

No doubt surgical residency poses a burden on the mental health of surgical trainees. However several limitations exist in our study. Firstly, a larger sample size is required in order to ensure a more accurate representation. In Kuwait, our surgical residency programs are relatively small, resulting in a smaller study cohort. A wider range of surgical specialties is also needed, however our local surgical residency program is limited in the range of surgical subspecialties. The results of the study might not be generalizable to surgical residents across different continents due to differences in cultural aspects, working hours, duration of residency training and variation in responsibilities. Several other variables can be included in a further study such as work relationships, perception of gender discrimination, career satisfaction, assessment of interpersonal skills and financial aspects. Finally a different study design can be employed to draw stronger conclusions e.g prospective cohort studies.

## 5 Conclusion

Surgical residents are a population at high risk for burnout. Ultimately, this may compound into patient care and lowers the quality of life for residents. Addressing burnout at all stages during residency is paramount in improving standard of care as well as increasing wellness of residents. In Kuwait targeted approaching should be implemented more thoroughly amongst our surgical residents – both for the sake of our patients and residents.

## Ethical approval

Ethical approval was obtained from the ethical committee at the ministry of health Kuwait.

## Sources of funding

This research did not receive any funding/grant from funding agencies in the public, commercial, or non-for-profit sectors.

## Author contributions

Waleed Burhamah (Corresponding author)- Study design, questionnaire design, literature review, writing of the first draft, final editing.

Abdulaziz AlKhayyat: literature review, writing of the first draft.

Melinda Oroszlányová: writing of the first draft and statistics.

Hana Jafar: Administering the questionnaire and writing of the first draft.

Ali AlKhayat: Administering the questionnaire and writing of the first draft.

Jasim Alabbad: research supervision, editing the final draft.

## Trail registry number

Name of the registry: clinicaltrials.gov NCT04808635.

Unique Identifying number or registration ID: MOHKWMKH21.

Hyperlink to your specific registration (must be publicly accessible and will be checked): https://clinicaltrials.gov/ct2/show/NCT04808635.

Will be publicly available within 2–5 business days.

## Guarantor

Waleed Burhamah.

## Consent

Informed consent was obtained from the responders for the publication of their responses anonymously.

## Provenance and peer review

Not commissioned, externally peer-reviewed.

## Declaration of competing interest

The authors declare no conflict of interest.
